# Mitochondrial CircRNA CircMT‐RNR2 Safeguards Antioxidant Defense to Support Fibroblast Functions in Wound Repair

**DOI:** 10.1002/advs.202517141

**Published:** 2026-02-08

**Authors:** Guanglin Niu, Jennifer Geara, Yongjian Chen, Lihua Luo, Yanwei Xiao, Zhuang Liu, Margaux Gaborieau, Ling Pan, Edmund Loh, Dongqing Li, Pehr Sommar, Aoxue Wang, Xiaowei Zheng, Ning Xu Landén

**Affiliations:** ^1^ Dermatology and Venereology Division Department of Medicine (Solna) Center For Molecular Medicine Karolinska Institute Stockholm Sweden; ^2^ Department of Dermatology The Second Hospital of Dalian Medical University College of Integrative Medicine Dalian Medical University Dalian China; ^3^ Department of Microbiology Tumor and Cell Biology Karolinska Institutet Solna Sweden; ^4^ Jiangsu Provincial Key Laboratory of Dermatology Hospital for Skin Diseases Institute of Dermatology Chinese Academy of Medical Sciences & Peking Union Medical College Nanjing China; ^5^ Clinical Microbiology Karolinska University Hospital Solna Sweden; ^6^ Singapore Centre on Environmental Life Sciences Engineering Nanyang Technological University Singapore Singapore; ^7^ Department of Plastic and Reconstructive Surgery Karolinska University Hospital Sweden and Department of Molecular Medicine and Surgery Karolinska Institute Stockholm Sweden; ^8^ Nordiska Kliniken Stockholm Sweden; ^9^ Department of Molecular Medicine and Surgery Karolinska Institutet Stockholm Sweden

**Keywords:** circRNA, DFU, mitochondria, wound healing

## Abstract

Diabetic foot ulcers (DFUs) are a debilitating diabetes complication in which mitochondrial dysfunction and oxidative stress are prominent but mechanistically unresolved features. Here, we identify the mitochondria‐encoded circular RNA (mecciRNA) circMT‐RNR2 as a novel modulator of mitochondrial redox homeostasis in human skin wound healing. CircMT‐RNR2 is reduced in DFU patient tissue and diabetic mouse wounds, enriched in dermal fibroblasts, and localized to mitochondria. Its loss impairs fibroblast proliferation, migration, extracellular matrix production, and contraction by destabilizing the mitochondrial antioxidant protein PRDX3, leading to elevated oxidative stress, mitochondrial damage, and mitophagy. In murine and human *ex vivo* wound models, circMT‐RNR2 knockdown delays healing, whereas overexpression accelerates repair and boosts antioxidant defenses. These findings position circMT‐RNR2 as a mitochondrial guardian of skin healing and a promising therapeutic target for DFU.

## Graphical abstract

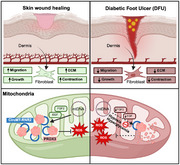

## 1 Introduction

Diabetes mellitus is a widespread chronic metabolic disorder with severe complications, among which diabetic foot ulcers (DFUs) are particularly debilitating [1]. DFUs are characterized by chronic inflammation, impaired vascularization, and stalled wound healing, often associated with elevated reactive oxygen species (ROS) levels that exacerbate tissue damage [2]. Despite medical advances, effective DFU therapies remain limited, highlighting the need to better understand their underlying mechanisms [3].

Mitochondria are central to cellular metabolism, ATP production, and redox signaling, and they represent the major source of intracellular ROS [4, 5]. Under physiological conditions, mitochondrial ROS (mtROS) act as signaling molecules; however, during mitochondrial stress or dysfunction, mtROS production and release become dysregulated. One key mechanism linking mitochondrial dysfunction to mtROS release is the opening of the mitochondrial permeability transition pore (mPTP), a nonspecific channel in the inner mitochondrial membrane that opens in response to elevated calcium levels and oxidative stress [6]. Sustained mPTP opening disrupts mitochondrial membrane potential and facilitates excessive mtROS release into the cytosol, thereby amplifying oxidative damage.

Mitochondrial redox balance is critical for wound repair, as controlled ROS signaling promotes fibroblast proliferation, extracellular matrix (ECM) synthesis, and tissue remodeling, whereas excessive ROS drives oxidative damage, chronic inflammation, and impaired healing [7]. In DFUs, this balance is disrupted by mitochondrial dysfunction and compromised antioxidant defenses, including downregulation of peroxiredoxin III (PRDX3), which further amplifies oxidative stress and stalls tissue repair [8, 9, 10]. Restoring mitochondrial redox homeostasis therefore represents a promising strategy for improving outcomes in chronic wounds.

Circular RNAs (circRNAs), which are covalently closed single‐stranded RNAs, have emerged as key regulators of gene expression by modulating transcription and splicing, stabilizing mRNAs, influencing translation, and interfering with signaling pathways [11]. While most circRNAs originate from the nuclear genome, recent studies have identified circRNAs encoded by the mitochondrial genome (mecciRNAs) in animals [12, 13, 14]. Although their biogenesis and turnover remain unclear, mecciRNAs are increasingly recognized as regulators of mitochondrial function [15, 16, 17]. For example, mecciND1 and mecciND5 act as molecular chaperones to facilitate mitochondrial protein import [12]. MecciRNA SCAR binds ATP5B to inhibit mPTP opening and reduce ROS output, previously shown to influence conditions such as chronic lymphocytic leukemia and nonalcoholic steatohepatitis [18, 19]. Given the central role of mitochondrial dysfunction in DFUs, we hypothesized that mecciRNAs may also contribute to diabetic wound pathogenesis.

Here, we identify circMT‐RNR2, a mecciRNA downregulated in DFUs, as a critical regulator of fibroblast function and wound repair. CircMT‐RNR2 maintains mitochondrial redox homeostasis by interacting with and stabilizing the antioxidant protein PRDX3, thereby limiting ROS‐induced damage and promoting fibroblast proliferation, extracellular matrix deposition, and tissue contraction. These findings reveal a novel link between mecciRNAs and redox control in diabetic wound healing, highlighting a promising therapeutic target for chronic wounds.

## 2 Results

### 2.1 CircMT‐RNR2 Expression is Downregulated in Diabetic Foot Ulcer

**FIGURE 1:**
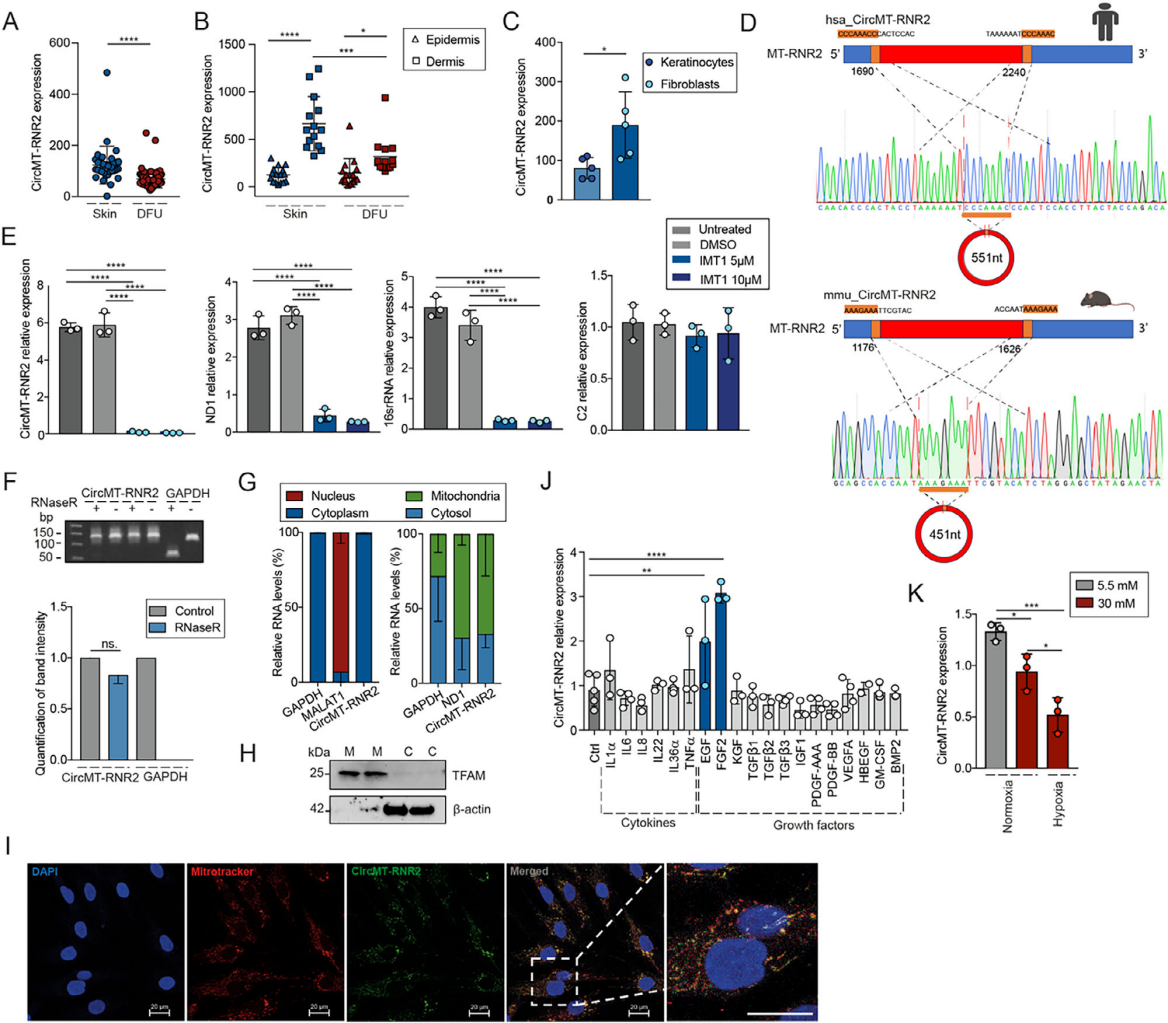
CircMT‐RNR2 expression is downregulated in diabetic foot ulcer. qRT‐PCR of circMT‐RNR2 expression in full‐thickness skin biopsies from healthy donors (n = 35) and diabetic foot ulcer (DFU) patients (n = 65) (A), and in the epidermal and dermal layers of skin (n = 16) and DFU samples (n = 16) (B). (C) qRT‐PCR analysis of circMT‐RNR2 expression in Keratinocytes and Fibroblasts isolated from human skin (n = 5). (D) Genomic location and junction sites of circMT‐RNR2. Sanger sequencing confirms human (up) and mouse (down) sequences; flanking repeats in orange. (E) qRT‐PCR of circMT‐RNR2, mitochondrial genes (*ND1*, 16S rRNA), and nuclear gene (C2) after POLRMT inhibition (IMT1, 5 or 10 µm, n = 3). (F) Agarose gel electrophoresis of circMT‐RNR2 and *GAPDH* RT‐PCR products from RNase R–treated vs. control RNA in HDFa cells; Bar graph shows band quantification. (G) qRT‐PCR of circMT‐RNR2, *GAPDH*, *MALAT1* and *ND1* in nuclear, cytoplasmic, mitochondrial and cytosolic fractions of HDFa cells (n = 2). (H) Western blot of mitochondrial and cytosolic fractions confirming purity (TFAM: mitochondrial marker; β‐actin: cytosolic marker). M = mitochondria, C = cytosol. (I) Fluorescence In Situ Hybridization analysis of circMT‐RNR2 and Mitotracker Red in human dermal fibroblasts, with nuclei counterstained by DAPI. Scalebar = 20 µm. (J) qRT‐PCR of circMT‐RNR2 in HDFa treated with cytokines or growth factors (24 h, n = 3–5). (K) circMT‐RNR2 expression in HDFa under low (5.5 mm) or high (30 mm) glucose, with or without hypoxia (n = 3). ns: not significant, ^*^ *P* < 0.05, ^**^ *P* < 0.01, ^***^ *P* < 0.001, ^****^ *P* < 0.0001, paired Student t‐test, or unpaired Student t‐test, or one‐way ANOVA and Turkey's multiple comparisons test.

From our previous RNA‐sequencing analysis of circRNAs in human skin and wound tissues, we identified a set of mecciRNAs [20]. Among these, circMT‐RNR2, a circular RNA derived from the mitochondrial gene MT‐RNR2 (encoding the large subunit of the mitochondrial ribosome 16s rRNA), was significantly downregulated in DFU tissue (n = 65) compared with normal human skin (n = 35), as confirmed by qRT‐PCR (Figure 1A; Table S1). In both skin and DFU samples, circMT‐RNR2 expression was higher in the dermis than in the epidermis (Figure 1B), consistent with its enrichment in dermal fibroblasts relative to epidermal keratinocytes (Figure 1C). Absolute qRT‐PCR further revealed that circMT‐RNR2 was present at 1473 ± 75 copies per fibroblast (Figure S1A). Based on these findings, we focused subsequent experiments on human adult dermal fibroblasts (HDFa) to investigate the functional role of circMT‐RNR2.

The junction site of circMT‐RNR2 was validated by Sanger sequencing. As reported for other mecciRNAs [12, 14], we identified repetitive sequence motifs flanking the junction, CCCAAAC in human and AAAGAAA in mouse, potentially involved in mecciRNA biogenesis (Figure 1D). The mitochondrial origin of circMT‐RNR2 was supported by the observation that treatment of HDFa with IMT1, a mitochondrial RNA polymerase inhibitor, significantly reduced circMT‐RNR2 abundance. This decrease paralleled reductions in mitochondrial RNAs (ND1 and 16S rRNA) but not the nuclear transcript C2 (Figure 1E). The circular structure of circMT‐RNR2 was further confirmed by its resistance to RNase R digestion, which degrades linear but not circular RNAs, as demonstrated by qRT‐PCR (Figure 1F) and Northern blot analysis (Figure S1B) [21].

To determine the subcellular localization of circMT‐RNR2, HDFa were fractionated into nuclear, cytoplasmic, and mitochondrial compartments. Enrichment of MALAT1 (nuclear), GAPDH (cytoplasmic), and ND1 (mitochondrial) confirmed the efficiency of the fractionation (Figure 1G), which was further validated at the protein level by Western blot analysis using TFAM (mitochondrial) and β‐actin (cytosolic) as compartment‐specific markers (Figure 1H). These analyses showed that circMT‐RNR2 is predominantly localized to mitochondria in human dermal fibroblasts (Figure 1G). This mitochondrial enrichment was further confirmed by fluorescence in situ hybridization (FISH), which revealed colocalization of circMT‐RNR2 with MitoTracker Red, a mitochondrial marker (Figure 1I).

We next investigated wound microenvironmental factors that might regulate circMT‐RNR2 expression. Treatment of HDFa with cytokines and growth factors essential for wound repair revealed that Epidermal Growth Factor (EGF) and Fibroblast Growth Factor 2 (FGF2) significantly increased circMT‐RNR2 levels (Figure 1J). In contrast, high glucose and hypoxic conditions reduced its expression (Figure 1K). Together, these findings suggest that the reduced circMT‐RNR2 observed in DFU may result from a combination of low EGF/FGF2 levels [22, 23], hyperglycemia, and hypoxia characteristic of this chronic wound type.

### 2.2 Loss of circMT‐RNR2 Impairs Fibroblast Functions Critical for Wound Healing

We next examined the role of circMT‐RNR2 in fibroblast‐driven wound repair processes, including proliferation, migration, contraction, and ECM production [24]. Since mitochondria harbor RNA interference machinery [25, 26, 27] and siRNA‐mediated knockdown of mitochondrial circRNAs has been demonstrated [27], we silenced circMT‐RNR2 in HDFa cells using siRNAs targeting its junction site (si‐CircMT‐RNR2). qRT‐PCR confirmed efficient mitochondrial knockdown of circMT‐RNR2 without affecting linear MT‐RNR2 expression (Figure S1C–E).

**FIGURE 2:**
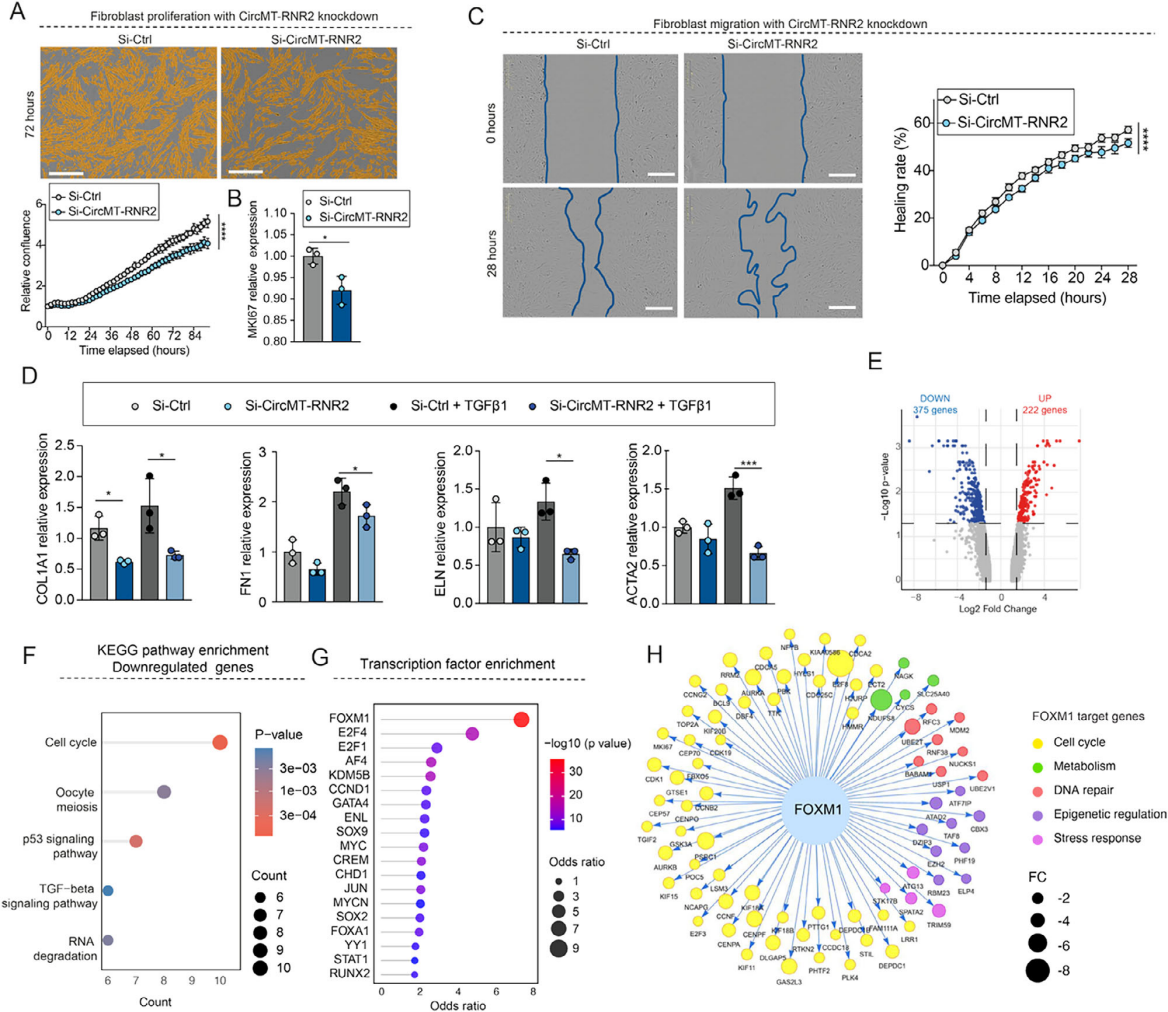
Loss of circMT‐RNR2 impairs fibroblast functions critical for wound healing. (A) Proliferation assay of HDFa cells treated with si‐Ctrl or si‐circMT‐RNR2 with quantification of relative confluence (n = 6); scale bar, 400 µm. (B) *MKI67* expression in HDFa cells with circMT‐RNR2 knockdown (microarray analysis, n = 3). (C) Scratch‐wound migration assay over 28 h in HDFa cells with si‐Ctrl or si‐circMT‐RNR2, with quantification of wound closure rate (%); scale bar, 400 µm. (D) qRT‐PCR of *COL1A1*, *FN1*, *ELN* and *ACTA2* in HDFa with Si‐Ctrl or Si‐CircMT‐RNR2, with or without TGF‐β1 stimulation (24 h, n = 3). (E) Volcano plot of differentially expressed genes between si‐Ctrl and si‐CircMT‐RNR2 (375 downregulated in blue, 222 upregulated in red; thresholds: FDR < 0.05, |log_2_FoldChange| > 1.5). KEGG pathway (F) and transcription factor (G) enrichment of downregulated genes. (H) FOXM1 target genes; node color indicates functional categories, node size reflects Fold Change (FC). ^*^ *p* < 0.05, ^**^ *p* < 0.01, ^***^ *p* < 0.001, ^****^ *p* < 0.0001 (unpaired Student t‐test, or two‐way ANOVA and multiple comparisons).

Knockdown of circMT‐RNR2 impaired several fibroblast functions critical for wound repair. Incucyte live‐cell imaging revealed a marked reduction in proliferation, which was further supported by decreased expression of the proliferation marker MKI67 (Figure 2A,B). In scratch‐wound assays, circMT‐RNR2–deficient fibroblasts displayed a small but significantly delayed migration compared to controls (Figure 2C). Furthermore, circMT‐RNR2 knockdown blunted TGF‐β1–induced expression of ECM genes (COL1A1, FN1, ELN) as well as ACTA2, which encodes α‐SMA, a key driver of fibroblast contractility (Figure 2D).

Transcriptomic microarray analysis revealed 375 downregulated and 222 upregulated genes following circMT‐RNR2 knockdown (FDR < 0.05, |log_2_FoldChange| > 1.5) (Figure 2E; Table S2). KEGG pathway enrichment showed that the cell cycle was the most significantly affected pathway among the downregulated genes (Figure 2F; Table S3). Transcription factor (TF) enrichment analysis further identified FOXM1 as the top upstream regulator of these genes, with most FOXM1 targets belonging to the cell cycle pathway (Figure 2G,H; Tables S4 and S5). Given that FOXM1 is a well‐established regulator of fibroblast migration, oxidative stress response, inflammation, and wound repair, and is implicated in DFU pathology [28, 29, 30], these findings suggest that circMT‐RNR2 supports fibroblast function at least in part by maintaining FOXM1‐driven transcriptional programs. Overall, circMT‐RNR2 emerges as a critical regulator of fibroblast proliferation, motility, ECM production, and contraction, thereby promoting effective wound healing.

### 2.3 CircMT‐RNR2 Safeguards Mitochondrial Redox Homeostasis

**FIGURE 3:**
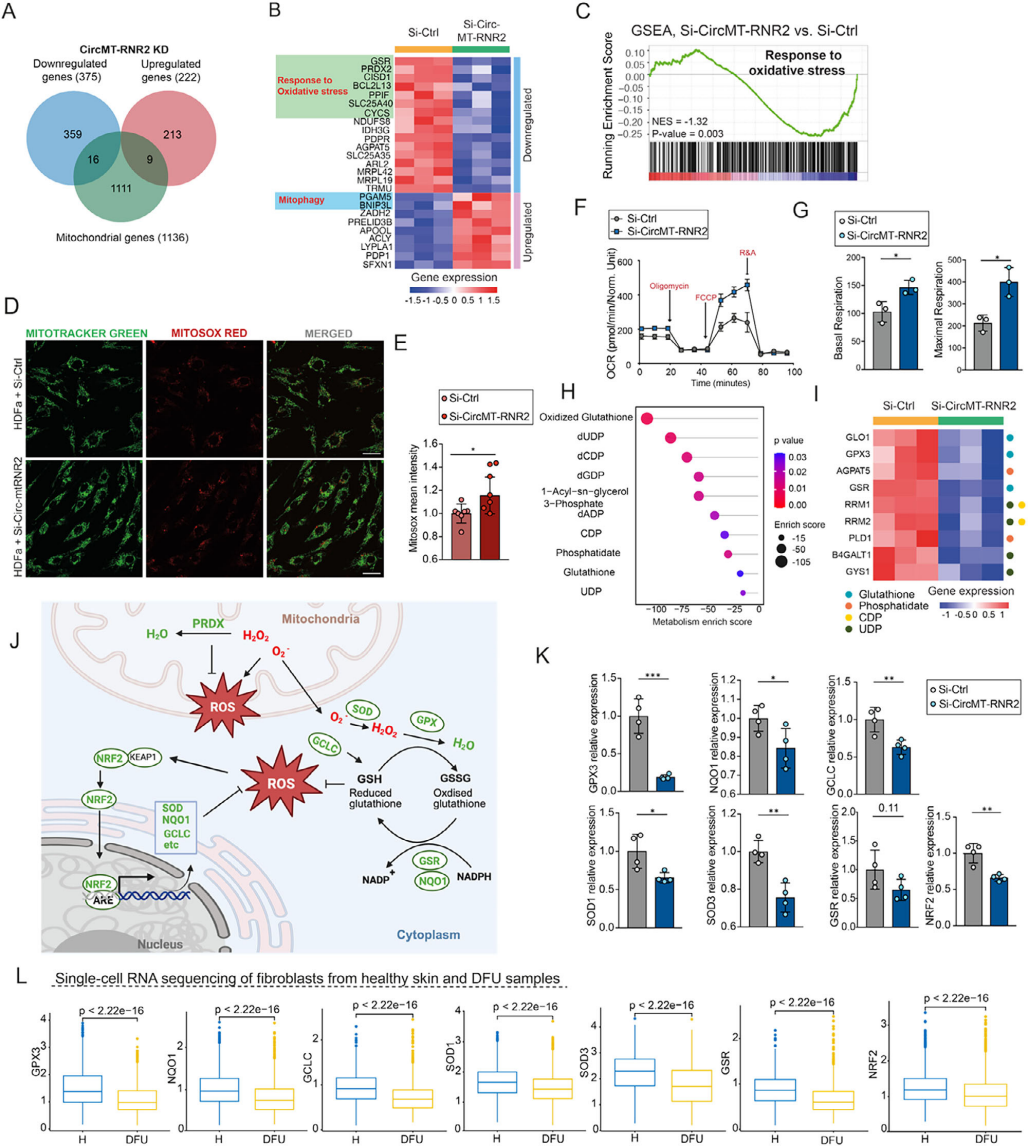
CircMT‐RNR2 safeguards mitochondrial redox homeostasis. (A) Venn diagram showing overlap between differentially expressed genes in HDFa with circMT‐RNR2 knockdown (KD) and mitochondrial genes. (B) Heatmap of up‐ and downregulated mitochondrial genes and their functions in HDFa with circMT‐RNR2 KD. (C) Gene Set Enrichment Analysis (GSEA) of oxidative stress response–related genes following circMT‐RNR2 KD. (D) Representative confocal images of HDFa cells stained with MitoTracker and MitoSOX after si‐Ctrl or si‐CircMT‐RNR2 treatment; scale bar, 30 µm (n = 7). (E) Quantification of MitoSOX mean fluorescence intensity. (F‐G) Seahorse analysis of mitochondrial respiration showing oxygen consumption rate (OCR), basal, and maximal respiration in HDFa with circMT‐RNR2 KD (n = 3). (H‐I) Metabolomic pathway analysis of genes downregulated upon circMT‐RNR2 knockdown and corresponding heatmap of affected metabolic pathway–associated genes. (J) Schematic of antioxidant defense and ROS homeostasis. (K) qRT‐PCR of antioxidant genes (GPX3, NQO1, GCLC, SOD1, SOD3, GSR, NRF2) in HDFa cells with circMT‐RNR2 KD (n = 4). (L) Single‐cell RNA‐sequencing analysis of the same antioxidant genes in dermal fibroblasts from healthy skin (H) (8425 cells from 9 donors) and DFU samples (10692 cells from 11 donors). ^*^ *p* < 0.05, ^**^ *p* < 0.01, ^***^ *p* < 0.001 (unpaired Student t test, or Mann–Whitney U test).

Given its mitochondrial origin and localization, we compared the microarray dataset following circMT‐RNR2 knockdown to the human MitoCarta3.0 database. Transcripts for 16 nuclear‐encoded mitochondrial proteins were downregulated while 9 were upregulated (Figure 3A) [31]. Notably, downregulated genes included GSR, PRDX2, CISD1, BCL2L13, PPIF, SLC25A40, and CYCS, which were all involved in the response to oxidative stress pathway [32, 33, 34, 35, 36, 37, 38] (Figure 3B). Consistent with this, gene set enrichment analysis (GSEA) revealed significant enrichment of the oxidative stress response pathway among downregulated genes (Figure 3C).

To directly assess oxidative stress, we co‐stained HDFa with MitoTracker Green (mitochondria) and MitoSOX Red (mitochondrial superoxide). CircMT‐RNR2 knockdown increased mitochondrial ROS levels, indicating elevated oxidative stress (Figure 3D,E). Similar ROS elevation was observed under hyperglycemic conditions, suggesting that circMT‐RNR2 deficiency mimics hyperglycemia‐induced stress (Figure S2A,B). Seahorse analysis further showed that circMT‐RNR2 knockdown increased oxygen consumption rate (OCR), as well as basal and maximal respiration (Figure 3F,G). Interestingly, enhanced mitochondrial respiration coupled with elevated ROS has been reported in chronic diabetic complications, including DFU, where it impairs fibroblast function and wound healing [39, 40, 41].

Given the pronounced increase in mitochondrial ROS, we next examined whether mPTP regulation was affected. Compared with HDFa treated with si‐Ctrl, circMT‐RNR2 knockdown resulted in significantly reduced Calcein AM fluorescence, indicating that a larger proportion of mitochondria remained in an open mPTP state (Figure S2C). Sustained mPTP opening is known to exacerbate mitochondrial dysfunction by promoting loss of membrane potential and facilitating mitochondrial ROS release [42], suggesting that increased mPTP opening contributes to the elevated oxidative stress observed following circMT‐RNR2 depletion.

Metabolic pathway analysis of the 375 genes downregulated upon circMT‐RNR2 knockdown revealed that circMT‐RNR2 silencing affected the oxidized glutathione pathway, indicating oxidative stress–related damage (Figure 3H) [43]. This was associated with loss of GSR, essential for glutathione recycling [44], and GLO1, which detoxifies harmful glycolysis byproducts [45] (Figure 3H–J). Reduced GLO1 activity promotes advanced glycation end‐product (AGE) accumulation in diabetic models, a known driver of diabetic complications [46]. Another key antioxidant gene, GPX3, was also downregulated; as a potent plasma peroxidase, GPX3 protects cells by reducing hydrogen peroxide and lipid hydroperoxides (Figure 3I,J) [47]. qRT‐PCR confirmed reduced expression of GPX3, GSR and additional antioxidants, including NRF2, NQO1, GCLC, SOD1 and SOD3, upon circMT‐RNR2 silencing (Figure 3K). NRF2 is the master regulator of the antioxidant response, translocating to the nucleus under oxidative stress to induce enzymes such as NQO1 (quinone detoxification), GCLC (glutathione synthesis), and SOD1/3 (superoxide dismutation in cytoplasm and extracellular space), which together form a coordinated defense network that maintains redox balance and protects cells from oxidative damage (Figure 3J) [48, 49, 50, 51, 52]. Importantly, re‐analysis of published single‐cell RNA‐sequencing data from healthy skin (n = 9) and DFU wound‐edge tissue (n = 11) revealed consistent downregulation of these antioxidant genes in dermal fibroblasts from DFU compared with non‐diabetic skin, underscoring the clinical relevance of circMT‐RNR2 deficiency (Figure 3L) [53].

CircMT‐RNR2 silencing also upregulated PGAM5 and BNIP3L, genes involved in mitophagy [54, 55, 56, 57] (Figure 3B). Western blotting confirmed increased levels of NIX (a mitophagy receptor) [55] and SIRT3 (a mitophagy activator) [58], alongside decreased TOMM20 (a mitochondrial integrity marker) [59], indicating enhanced mitophagy and mitochondrial damage (Figure S2D). Analysis of global autophagic activity revealed that circMT‐RNR2 knockdown increased LC3‐I and LC3‐II accumulation after ammonium chloride (NH_4_Cl) treatment, which inhibits lysosomal degradation by neutralizing acidity and causes autophagosome accumulation [60, 61]. This demonstrates elevated autophagic flux following circMT‐RNR2 silencing (Figure S2E).

Collectively, these data indicate that circMT‐RNR2 maintains mitochondrial redox homeostasis not only by sustaining antioxidant defenses but also by limiting ROS‐sensitive mPTP opening. Its loss promotes oxidative stress, mitochondrial dysfunction, mPTP opening, and compensatory mitophagy, thereby mimicking diabetic‐related cellular dysfunction.

### 2.4 CircMT‐RNR2 Interacts With and Stabilizes PRDX3

**FIGURE 4:**
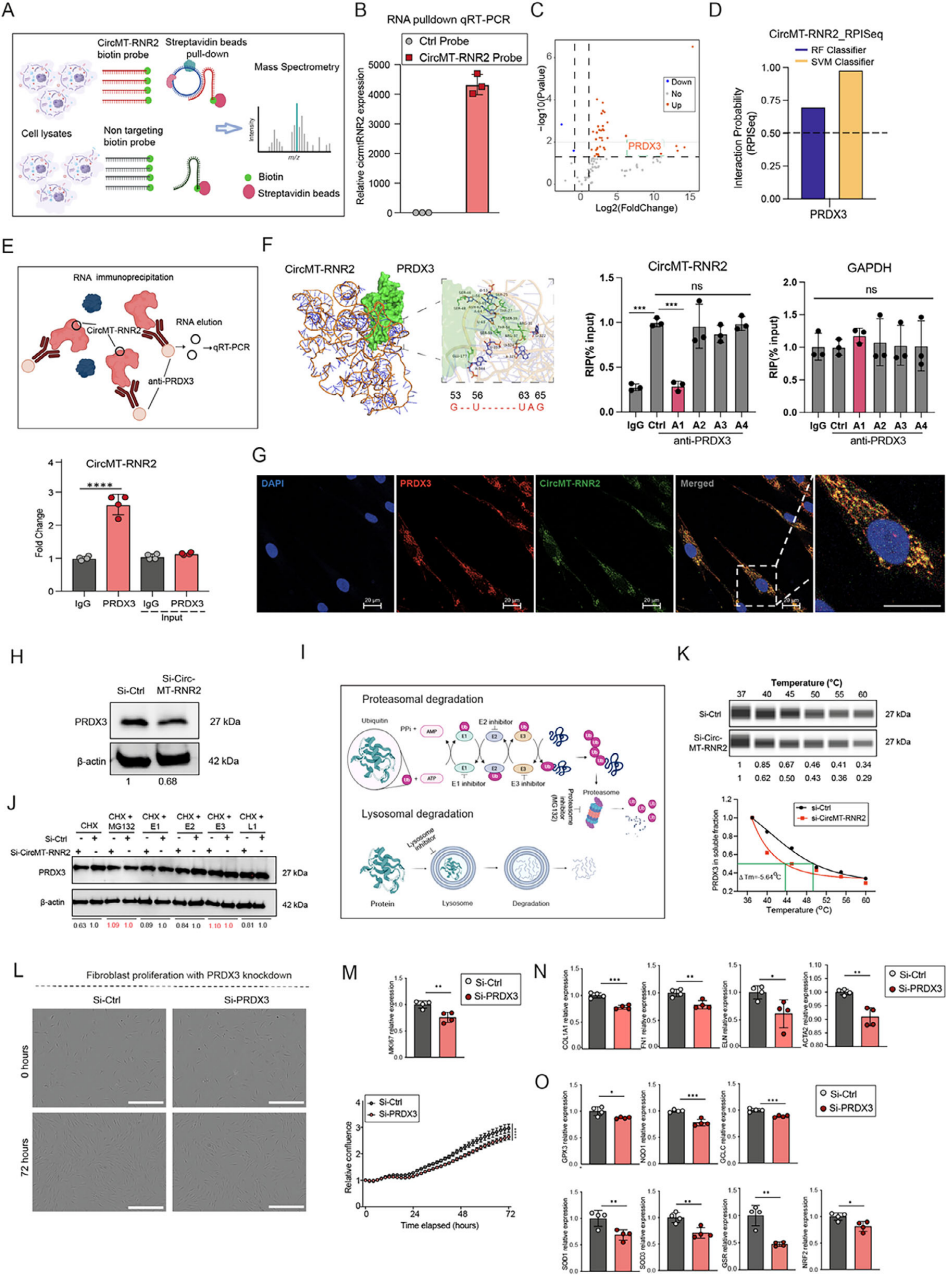
CircMT‐RNR2 interacts with and stabilizes PRDX3. (A) Schematic of RNA pulldown using biotin‐labeled circRNA probes and streptavidin beads followed by mass spectrometry (MS). (B) qRT‐PCR validation of circMT‐RNR2 enrichment after pulldown (n = 3). (C) Volcano plot of proteins identified by MS. (D) RPISeq prediction showing strong interaction potential between PRDX3 and circMT‐RNR2. (E) RNA immunoprecipitation (RIP) schematic (upper) and qRT‐PCR detection of circMT‐RNR2 in lysates immunoprecipitated with anti‐PRDX3 or IgG control (n = 4, lower). (F) Predicted structure of the circMT‐RNR2‐PRDX3 complex with a zoomed‐in view of the binding interface (left panel). qRT‐PCR analysis of circMT‐RNR2 and GAPDH mRNA in RNPs immunoprecipitated using anti‐PRDX3 antibodies or IgG from fibroblasts transfected with antisense oligos (A1‐A4) targeting potential PRDX3 binding sites in circMT‐RNR2 RNA (n = 3, right panel). (G) Combined Fluorescence In Situ Hybridization and Immunofluorescence analysis of circMT‐RNR2 and PRDX3 protein in human dermal fibroblasts, with nuclei counterstained by DAPI. Scalebar = 20 µm. (H) Western blot of PRDX3 in HDFa cells with circMT‐RNR2 knockdown; β‐actin as control. (I) Schematic of protein degradation pathways. (J) Western blot of PRDX3 in circMT‐RNR2–depleted fibroblasts treated with CHX alone or in combination with proteasomal (E1–E3) or lysosomal (L1) inhibitors; quantification relative to β‐actin. (K) CETSA assay of PRDX3 stability: Simple Western (upper) and melting curves (lower) in HDFa cells with circMT‐RNR2 silencing. (L) Proliferation assay of HDFa cells with PRDX3 knockdown and quantification of cell confluence (72 h, n = 6); scale bar, 400 µm. (M) qRT‐PCR of *MKI67* after PRDX3 knockdown (n = 4). (N) qRT‐PCR of *COL1A1, FN1, ELN*, and *ACTA2* in HDFa cells with PRDX3 knockdown (n = 4). (O) qRT‐PCR of antioxidant genes (GPX3, NQO1, GCLC, SOD1, SOD3, GSR, NRF2) after PRDX3 knockdown (n = 4). ^*^P <0.05, ^**^ *P* < 0.01, ^***^ *P* < 0.001, ^****^ *P* < 0.0001 (unpaired Student t.test, or two‐way ANOVA and multiple comparisons).

To investigate the molecular mechanism of circMT‐RNR2, we profiled its protein interactome using RNA pulldown in HDFa cells (Figure 4A) [62]. A biotin‐labeled probe targeting the junction site of circMT‐RNR2 enriched circMT‐RNR2 over 4,000‐fold compared with a control probe, as confirmed by qRT‐PCR (Figure 4B). Silver staining revealed more proteins pulled down by the circMT‐RNR2 probe than by the control (Figure S3A). Label‐free mass spectrometry identified 37 proteins significantly enriched with the circMT‐RNR2 probe (Figure 4C; Table S7). Although the pulldown used whole‐cell lysate, 5 of the top 11 enriched proteins were mitochondrial, supporting a mitochondrial localization and function for this circRNA. Among these, Peroxiredoxin 3 (PRDX3), a mitochondria‐specific ROS scavenger, drew particular attention given circMT‐RNR2's role in mitochondrial redox balance [63]. RNA‐Protein Interaction Prediction (RPISeq) analysis predicted a strong circMT‐RNR2–PRDX3 interaction, with both RF and SVM classifiers scoring above the 0.5 binding threshold (Figure 4D) [64]. RNA immunoprecipitation (RIP) with PRDX3 antibody confirmed this interaction: circMT‐RNR2 was significantly enriched in the PRDX3 immunoprecipitate compared with the IgG control (Figure 4E).

Moreover, we used Chai‐1 (AlphaFold3) [65] to predict the 3D structure of the circMT‐RNR2–PRDX3 complex and analyzed putative PRDX3‐binding regions within circMT‐RNR2 using PyMOL (Figure 4F) [66]. To validate these in silico predictions, we designed four antisense oligonucleotides (ASOs; sequences and target regions are listed in Table S8) targeting the predicted PRDX3‐binding sites and transfected them into human dermal fibroblasts. RIP assays showed that PRDX3 failed to pull down circMT‐RNR2 when ASO1, which blocks nucleotides 53–65 relative to the BSJ, was used, whereas the other ASOs had no comparable effect. These results indicate that this region is critical for PRDX3 binding (Figure 4F). Additionally, cytoplasmic colocalization of circMT‐RNR2 RNA and PRDX3 protein, observed by combined fluorescence in situ hybridization (FISH) and immunofluorescence (IF) analysis, further supports a direct interaction between circMT‐RNR2 and PRDX3 (Figure 4G).

Furthermore, we found that PRDX3 protein levels decreased upon circMT‐RNR2 knockdown (Figure 4H). To assess whether circMT‐RNR2 regulates PRDX3 production or degradation, fibroblasts were treated with cycloheximide (CHX) to block translation, in combination with lysosomal or proteasomal inhibitors to prevent protein degradation (Figure 4I) [67]. Blocking degradation, but not translation, restored PRDX3 levels in circMT‐RNR2‐depleted cells, suggesting that circMT‐RNR2 stabilizes PRDX3 protein (Figure 4J). This was supported by Cellular Thermal Shift Assay (CETSA), where circMT‐RNR2 silencing reduced PRDX3 melting temperature, indicating decreased protein stability (Figure 4K) [68]. Interestingly, reciprocal regulation was observed, as qRT‐PCR showed that PRDX3 depletion significantly reduced CircMT‐RNR2 levels in human dermal fibroblasts (Figure S3B,C), indicating that the circMT‐RNR2–PRDX3 interaction helps maintain the abundance of both binding partners.

PRDX3 is essential for mitochondrial function and oxidative stress protection [63]. Its knockdown increases mitochondrial ROS, disrupts mitochondrial function, and impairs cancer cell proliferation [69, 70, 71]. In fibroblasts, we showed that PRDX3 silencing (Figure S3B) reduced cell proliferation and MKI67 expression – a proliferation marker (Figure 4L,M), decreased ECM gene expression (COL1A1, FN1, ELN), and lowered ACTA2 levels (Figure 4N). It also reduced the expression of antioxidant enzymes (GPX3, NQO1, GCLC, SOD1, SOD3, GSR) and the master antioxidant regulator NRF2 (Figure 4O). Thus, PRDX3 knockdown phenocopied circMT‐RNR2 depletion, impairing fibroblast proliferation, ECM production, contractility, and mitochondrial antioxidant defense. These results support a model in which circMT‐RNR2 binds and stabilizes PRDX3 to preserve mitochondrial redox homeostasis.

### 2.5 CircMT‐RNR2 is Essential for Wound Healing in Murine In Vivo Wound Model

**FIGURE 5:**
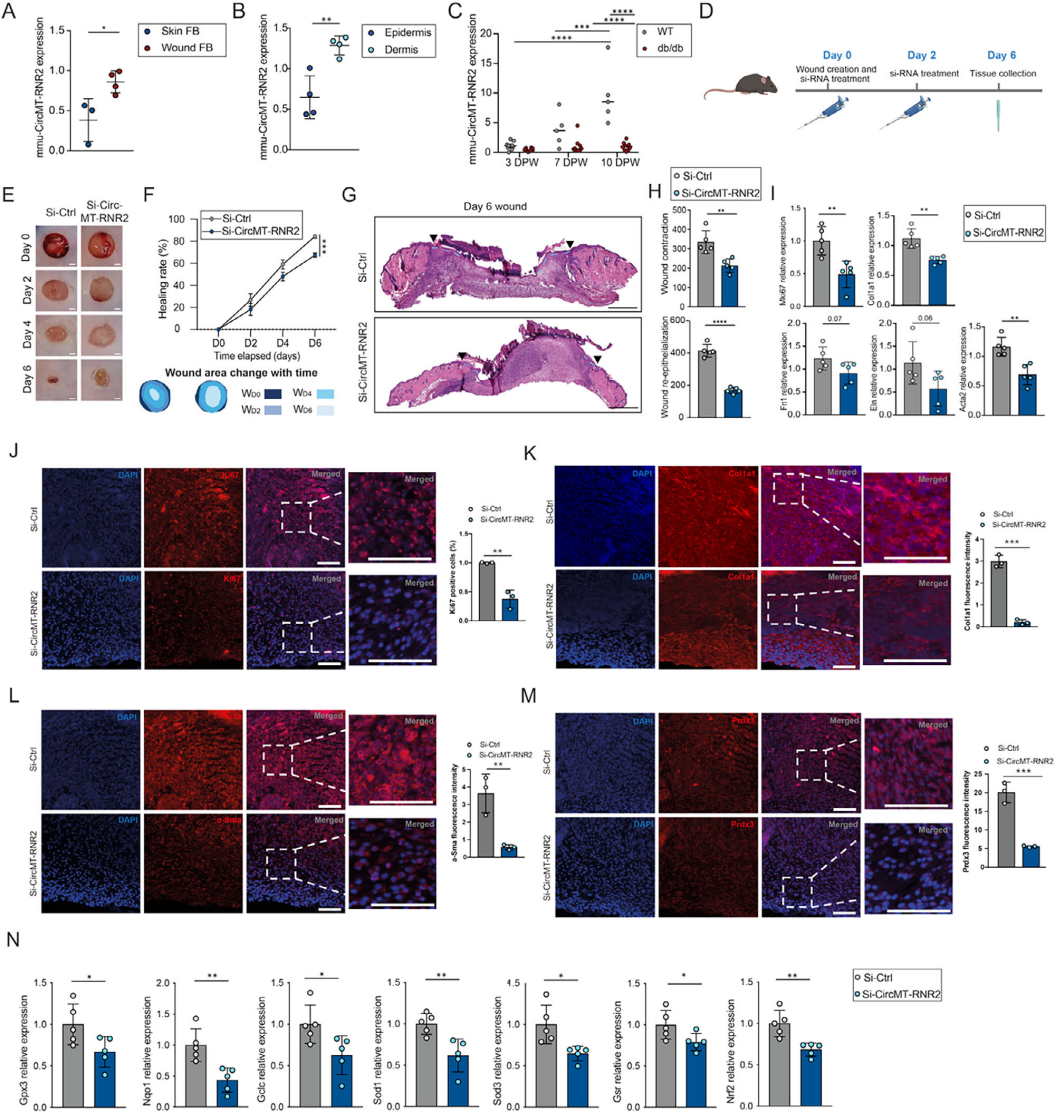
CircMT‐RNR2 is required for wound closure in vivo. qRT‐PCR analysis of mmu_circMT‐RNR2 in (A) mouse skin (n = 3) and wound fibroblasts (n = 4); (B) murine epidermis vs. dermis (n = 4); (C) during wound healing in wild‐type and db/b mice (days post‐wounding, DPW; n = 5‐10). (D) Schematic of topical siRNA treatment targeting mmu_circMT‐RNR2. (E) Macroscopic wound images (Day 0–Day 6) after siRNA treatment (n = 5). Scale bar = 1000 µm. (F) Quantification of wound healing rate (%) from (E). (G) Representative H&E images of D6 wounds after Si‐Ctrl or Si‐mmu‐CircMT‐RNR2 treatment (n = 5). Scale bar = 500 µm. Dashed blue lines mark new epidermis; arrowheads indicate initial wound edges. (H) Quantification of re‐epithelialization and contraction from (G). (I) qRT‐PCR of *Mki67*, *Col1a1*, *Fn1*, *Eln* and *Acta2* from D6 wound biopsies (n = 5). (J–M) Representative immunofluorescence images of mouse wounds stained for (J) Ki67, (K) Col1a1, (L) α‐Sma, and (M) Prdx3, with signal quantification (n = 3). Scale bar = 100 µm. (N) qRT‐PCR of *Gpx3*, *Nqo1*, *Gclc*, *Sod1*, *Sod*3, *Gsr* and *Nrf2* in mouse dermis after siRNA treatment (n = 5). *P < 0.05, ^**^ *P* < 0.01, ^***^ *P* < 0.001, ^****^P < 0.0001 (unpaired t‐test, or one‐way ANOVA and Turkey's multiple comparison test, or two‐way ANOVA and multiple comparisons).

Given that circMT‐RNR2 is conserved between humans and mice (Figure 1D), we examined its expression in a murine wound healing model. CircMT‐RNR2 was upregulated in wound fibroblasts compared with skin fibroblasts and was more abundant in dermis than epidermis, mirroring its distribution in human skin (Figure 5A,B). In wild‐type mice, circMT‐RNR2 levels increased progressively during wound healing, whereas in db/db mice, a type 2 diabetes model, its expression remained unchanged, consistent with reduced circMT‐RNR2 in human DFU (Figure 5C; Figure S4A, B) [72].

To test whether circMT‐RNR2 is required for wound repair, we topically applied si‐CircMT‐RNR2 or si‐Ctrl to dorsal skin wounds of wild‐type mice immediately after injury and again two days later, monitoring healing over six days (Figure 5D). Si‐CircMT‐RNR2 treatment significantly reduced dermal circMT‐RNR2 expression through day 6 (Figure S4C) without affecting body weight, indicating no systemic toxicity (Figure S4D). Macroscopically, circMT‐RNR2 knockdown delayed wound closure (Figure 5E,F). Hematoxylin and Eosin (H&E) staining of day 6 wounds confirmed reduced contraction and re‐epithelialization (Figure 5G,H). qRT‐PCR revealed lower expression of the proliferation marker Mki67, ECM genes (Col1a1, Fn1, Eln), and the contractility gene Acta2 (Figure 5I). Reduced Mki67, Col1a1, and Acta2 protein levels were confirmed by immunofluorescence (Figure 5J–L). Notably, circMT‐RNR2 knockdown markedly decreased Prdx3 protein in wound tissue (Figure 5M). In parallel, it suppressed the expression of antioxidant enzymes (Gpx3, Nqo1, Gclc, Sod1, Sod3, Gsr) and the master antioxidant regulator Nrf2 (Figure 5N).

Collectively, these results demonstrate that circMT‐RNR2 is critical for wound healing in vivo, acting through Prdx3 stabilization and the preservation of mitochondrial redox balance, thereby protecting cells from oxidative stress–induced damage.

### 2.6 CircMT‐RNR2 Promotes Healing of human Ex Vivo Wounds

**FIGURE 6:**
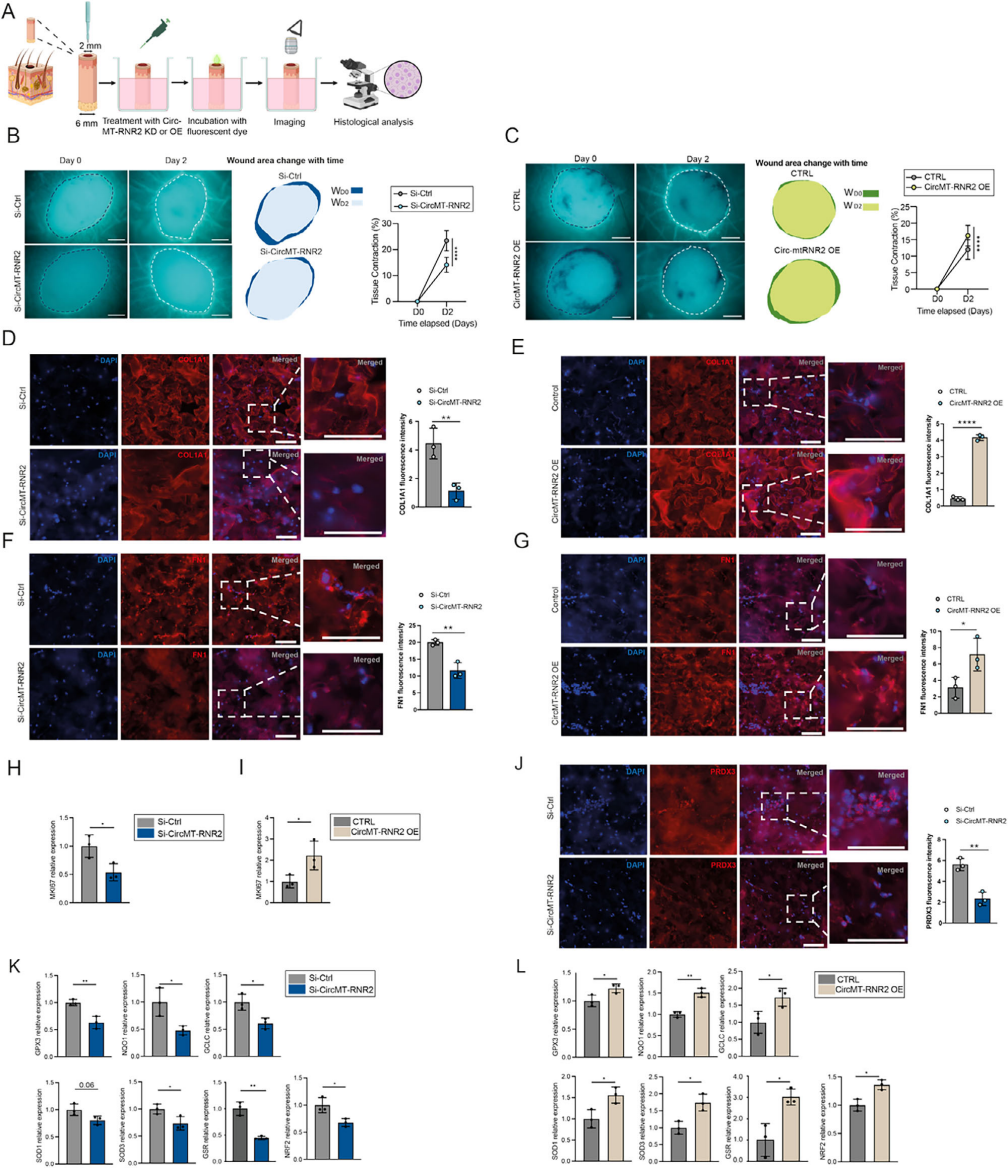
CircMT‐RNR2 promotes healing of human ex vivo wounds. (A) Schematic illustration of topical treatment of human *ex vivo* wounds with si‐RNA targeting circMT‐RNR2 (KD) or circMT‐RNR2 overexpression vector (OE). (B‐C) Fluorescent images of wound areas at D0 and D2. Wounds treated with Si‐Ctrl or Si‐CircMT‐RNR2 (B) and Control or circMT‐RNR2 OE (C) are shown (left); wound edges are indicated by dashed lines. Areas within the wound edge (IW) at each time point are illustrated (right). Scale bars: 500 µm. Wound contraction (%) was calculated as ΔIW_D2_ /IW_D0_×100% (n = 3/group). (D‐G) Representative immunofluorescence images showing COL1A1 (D, E) and FN1 (F, G) in wounds with circMT‐RNR2 KD (D, F) or OE (E, G) for 7 days. Scale bars: 100 µm (H‐I) qRT‐PCR analysis of *MKI67* in these treated human wounds. (J) Immunofluorescence images of PRDX3 in wounds with circMT‐RNR2 KD. (K‐L) qRT‐PCR analysis of oxidative stress‐related genes (*GPX3*, *NQO1*, *GCLC*, *SOD1*, *SOD3*, *GSR*, *NRF2)* in treated dermis (n = 3). ^*^P<0.05, ^**^ *P* < 0.01, ^***^ *P* < 0.001, ^****^ *P* < 0.0001 (unpaired Student t‐test, or two‐way ANOVA and multiple comparisons).

To evaluate the translational relevance of our findings, we investigated the role of circMT‐RNR2 in wound repair using a human ex vivo skin model (Figure 6A). In this system, wounds are introduced into surgically discarded human skin samples, which are then maintained under standard culture conditions. This approach preserves native tissue architecture, allowing the study of human skin repair in an environment that closely resembles in vivo conditions [73, 74].

We modulated circMT‐RNR2 levels in ex vivo wounds by silencing with si‐CircMT‐RNR2 (Figure S5A) or by overexpressing (OE) using a plasmid based on a previously established method for mitochondrial circRNA expression [27]. qRT‐PCR confirmed efficient mitochondrial overexpression of circMT‐RNR2 in human dermal fibroblasts without affecting MT‐RNR2 expression (Figure S5B–D), and successful circMT‐RNR2 overexpression in the wound dermis of the ex vivo model (Figure S5E).

Functionally, silencing circMT‐RNR2 impaired tissue contraction, whereas overexpression promoted it (Figure 6B,C). Immunofluorescence analysis revealed that knockdown of circMT‐RNR2 decreased dermal expression of COL1A1 and FN1, whereas overexpression increased their levels (Figure 6D–G). Similarly, silencing circMT‐RNR2 reduced, while overexpression increased, the proliferation marker MKI67 (Figure 6H,I). Notably, PRDX3 protein was reduced in circMT‐RNR2–silenced wounds, supporting circMT‐RNR2's role in stabilizing PRDX3 during human wound repair (Figure 6J). Moreover, qRT‐PCR revealed that circMT‐RNR2 knockdown suppressed antioxidant enzymes (GPX3, NQO1, GCLC, SOD1, SOD3, GSR) and the master antioxidant regulator NRF2, while overexpression enhanced their expression (Figure 6K,L).

To determine whether PRDX3 mediates the effects of circMT‐RNR2 in wound repair, we combined circMT‐RNR2 overexpression with PRDX3 knockdown (Figure S6A,B). PRDX3 silencing largely abolished the pro‐contractile effects induced by circMT‐RNR2 overexpression, indicating that circMT‐RNR2–driven tissue contraction requires PRDX3 (Figure S6C,D). Consistently, the circMT‐RNR2–induced increases in COL1A1 protein, proliferation marker MKI67, and antioxidant genes (GCLC, GSR, SOD1, SOD3, and NRF2) were markedly attenuated upon PRDX3 knockdown (Figure S6E,F). Together, these findings establish PRDX3 as a key downstream effector of circMT‐RNR2 and underscore circMT‐RNR2 as a central regulator of fibroblast‐driven skin repair with therapeutic potential.

## 3 Discussion

**FIGURE 7:**
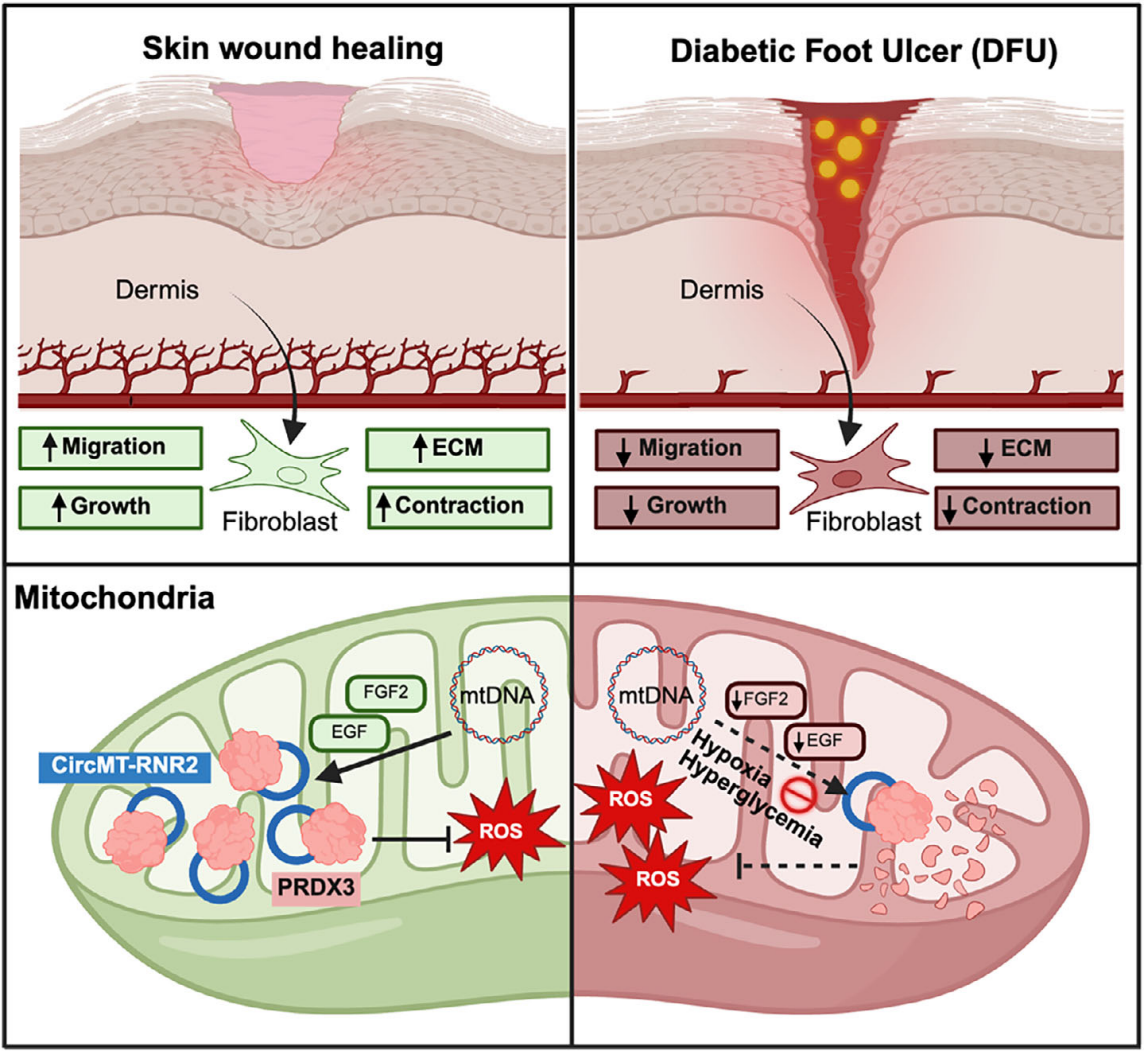
Schematic summary of the study findings. CircMT‐RNR2 promotes wound healing by enhancing fibroblast proliferation, migration, contraction, and extracellular matrix (ECM) production. It sustains mitochondrial redox balance by stabilizing the antioxidant protein PRDX3, thereby reducing ROS‐induced damage. In diabetic foot ulcers, hypoxia, hyperglycemia, and reduced FGF2 and EGF expression lower circMT‐RNR2 levels, impairing fibroblast function and delaying healing.

Mitochondria‐encoded circular RNAs (mecciRNAs) are a newly recognized class of non‐coding RNAs that expand our understanding of how mitochondria regulate cellular homeostasis and stress adaptation [75]. Although hundreds of mecciRNAs have been cataloged in human and murine cells, only a few have been functionally characterized, each revealing important roles in mitochondrial biology [12, 13, 14, 16]. Here, we identify circMT‐RNR2 as a novel functional mecciRNA essential for wound repair. By stabilizing the antioxidant protein PRDX3, circMT‐RNR2 safeguards mitochondrial redox homeostasis, thereby supporting fibroblast activity crucial for tissue repair. Its deficiency in DFUs compromises fibroblast function and delays healing, providing the first evidence that a pathophysiologically relevant mecciRNA contributes to human tissue repair (Figure 7).

Mitochondrial transcripts are synthesized as long polycistronic RNAs, then processed into mRNAs, tRNAs, and rRNAs primarily through tRNA‐guided cleavage by RNase P and RNase Z [76, 77, 78]. While the tRNA punctuation model explains much of this process, the existence of non–tRNA‐flanked transcripts and newly identified mitochondrial non‐coding RNAs suggests alternative RNA processing pathways [16]. Unlike nuclear circRNAs, which form through backsplicing, mecciRNAs are unlikely to use canonical splicing machinery, which is absent in mitochondria [12, 79]. Notably, circMT‐RNR2 and many other mecciRNAs harbor conserved repetitive sequences at their junctions that may facilitate mitochondrial RNA ligation (Figure 1C) [12, 14]. Although nuclear mitochondrial DNA segments could raise questions about circMT‐RNR2's genomic origin, our data show that inhibiting mitochondrial RNA polymerase reduces circMT‐RNR2 levels without affecting nuclear RNAs, supporting its mitochondrial origin (Figure 1D) [80]. These observations suggest that mecciRNAs arise via a unique and currently uncharacterized mitochondrial mechanism, warranting further investigation.

Our data show that circMT‐RNR2 expression in human dermal fibroblasts is dynamically regulated, upregulated by growth factors such as EGF and FGF2, but suppressed under hyperglycemic or hypoxic conditions. This regulation mirrors that of nuclear circRNAs, indicating that circMT‐RNR2 is not a transcriptional byproduct but a responsive regulatory molecule. In DFUs, loss of proliferating fibroblasts correlates with reduced EGF signaling [81], and diminished FGF2 further impairs fibroblast activation and angiogenesis [82]. Combined with the hostile metabolic and hypoxic microenvironment of DFUs, these deficiencies likely converge to suppress circMT‐RNR2 expression in chronic wounds.

Redox homeostasis is essential for wound healing, and our findings indicate that circMT‐RNR2 maintains mitochondrial ROS balance through complementary protective mechanisms. CircMT‐RNR2 directly binds and stabilizes PRDX3, a mitochondria‐specific ROS scavenger induced under oxidative stress [63, 83], thereby limiting mitochondrial ROS accumulation. In parallel, circMT‐RNR2 depletion increases mPTP opening, indicating impaired maintenance of mitochondrial membrane integrity. Sustained mPTP opening collapses membrane potential and promotes further mitochondrial ROS release, establishing a feed‐forward cycle of oxidative stress and mitochondrial dysfunction [42]. Because mitochondrial ROS sensitizes mPTP opening [84, 85, 86], PRDX3‐dependent ROS buffering may indirectly restrain pathological pore activation under stress. Together, these findings suggest that circMT‐RNR2 protects fibroblasts from oxidative injury by coordinately reinforcing antioxidant defense and limiting ROS‐driven mPTP opening in the hostile microenvironment of diabetic wounds. Consistent with this concept, recent studies report that additional mecciRNAs regulate mitochondrial ROS release and mPTP activity, supporting a broader role for mecciRNAs as modulators of mitochondrial stress signaling [19, 87].

FOXM1 may represent an additional link between mitochondrial redox status and nuclear transcriptional responses. FOXM1 is a well‐established regulator of oxidative stress programs whose expression and activity respond to ROS‐dependent signaling [88], and it transcriptionally upregulates PRDX3 to support mitochondrial function under stress [69]. CircMT‐RNR2 may therefore reinforce PRDX3 levels through both protein stabilization and transcriptional regulation, potentially via FOXM1. How circMT‐RNR2 influences FOXM1 expression or activity, however, remains to be determined and warrants further investigation.

In summary, this study identifies circMT‐RNR2 as a novel regulator of mitochondrial antioxidant defense, with direct relevance to tissue repair. Beyond establishing the first functional link between a mecciRNA and human wound healing, our findings broaden the understanding of mitochondrial RNA biology and highlight circMT‐RNR2 as a potential therapeutic target for chronic, non‐healing wounds in diabetes.

## 4 Materials and Methods

### 4.1 Human Skin Specimens

Human skin samples and DFU tissues were collected at the Second Hospital of Dalian Medical University, China (Table S1). DFU patients enrolled in the study had non‐healing ulcers persisting for >2 months despite conventional treatment. Tissue was obtained from the non‐healing wound edges using a 4‐mm biopsy punch. Human skin for cell isolation and ex vivo wound model experiments was sourced from discarded abdominal tissue following plastic surgery at the Department of Reconstructive Plastic Surgery, Karolinska University Hospital, Sweden (Table S1). All participants provided written informed consent. The study protocols were approved by the Ethics Committee of The Second Hospital of Dalian Medical University (2015‐102, 2016–28) and the Stockholm Regional Ethics Committee (2019‐02335), and conducted in accordance with the Declaration of Helsinki.

### 4.2 Microarray Analysis

Transcriptomic profiling was performed by employing the Affymetrix Human Clariom S Assay (Thermo Fisher Scientific) at the Bioinformatics and Expression Analysis (BEA) Core Facility, Karolinska Institutet. RNA quality and quantity were assessed using a NanoDrop 1000 spectrophotometer (Thermo Fisher Scientific) and an Agilent 2200 TapeStation with RNA ScreenTape (Agilent, Santa Clara, CA). A total of 150 ng RNA was used for cDNA synthesis according to the GeneChip WT PLUS Reagent Kit protocol (Thermo Fisher Scientific). Standard Affymetrix procedures, including hybridization, fluidics processing, and scanning, were followed. Genes exhibiting |log_2_FoldChange| >1.5 with FDR < 0.05 were considered differentially expressed (DEGs). Results were visualized as heatmaps and volcano plots generated using the “heatmap” and “ggplot2” R packages. Overlaps between DEGs and mitochondrial genes were illustrated with a Venn diagram. The dataset is available at the Gene Expression Omnibus under accession number GSE301636.

To investigate the biological pathways associated with DEGs, Kyoto Encyclopedia of Genes and Genomes (KEGG) pathway enrichment analysis was conducted using the R package clusterProfiler, applying a significance cutoff of p < 0.05. Additionally, Gene Set Enrichment Analysis (GSEA) was performed to compare functional differences between groups. Metabolomic enrichment and transcription factor prediction analyses were carried out using the Enrichr platform (https://maayanlab.cloud/Enrichr/) [89]. Lollipop plots depicting differences in metabolomic pathways and transcription factors between HDFa si‐Ctrl and HDFa si‐CircMT‐RNR2 groups were generated using the R package ggplot2.

### 4.3 Cell Culture and Treatments

Primary human adult dermal fibroblasts (HDFa, C0135C, contamination free; Cascade Biologics, Portland, OR) were cultured in Dulbecco's Modified Eagle Medium high glucose (DMEM, ThermoFisher Scientific) supplemented with 10% heat inactivated Fetal Bovine Serum (HI‐FBS) and 1% antibiotic cocktail (penicillin/ streptomycin) at 37°C in 5% CO_2_. To mimic hypoxia, cells were incubated under hypoxic conditions within a chamber set to 5% oxygen (O_2_).

To investigate factors potentially regulating circMT‐RNR2 expression, HDFa cells were treated with the following cytokines and growth factors: IL‐1α (20 ng/ml), IL‐6 (50 ng/ml), IL‐8 (50 ng/ml), IL‐22 (30 ng/ml), IL‐36α (100 ng/ml), TNF‐α (50 ng/ml), TGF‐β1 (20 ng/ml), TGF‐β2 (10 ng/ml), TGF‐β3 (20 ng/ml), BMP‐2 (100 ng/ml), EGF (20 ng/ml), IGF‐1 (20 ng/ml), FGF‐2 (30 ng/ml), VEGFA (20 ng/ml), HB‐EGF (20 ng/ml) or PBS as a control. Treatment was applied for 24 h, after which circMT‐RNR2 expression was analyzed by qRT‐PCR. All cytokines and growth factors were obtained from ImmunoTools (Friesoythe, Germany) or R&D Systems (Minneapolis, MN).

To inhibit mitochondrial transcription, cells were treated with 5 or 10 µm IMT1 (HY‐134539, MedChemExpress) for 200 hours to block mitochondrial RNA polymerase. The culture medium was replaced every other day with fresh medium containing the same concentration of IMT1.

To assess autophagy, HDFa cells transfected with Si‐Ctrl or Si‐CircMT‐RNR2 were treated with 50 nM ammonium chloride (NH_4_Cl; HY‐Y1269C, MedChemExpress) for 3 hours. Proteins were then extracted and analyzed by Western blot.

To study the functions of circMT‐RNR2/PRDX3 in fibroblasts, knockdown and overexpression experiments were performed. For knockdown, cells at 70% confluence were transfected with predesigned siRNA targeting circMT‐RNR2 (Si‐CircMT‐RNR2, Dharmacon) or with siRNA targeting PRDX3 (Si‐PRDX3, Dharmacon) and a non‐targeting control siRNA (Si‐Ctrl, Dharmacon) for 24 hours using RNAiMAX as transfection reagent, in nutrient depleted, antibiotic free medium. To overexpress circMT‐RNR2, fibroblasts at 80–90% confluence were transfected with circMT‐RNR2 overexpression plasmid using Lipofectamine 3000 for 24 hours.

To examine circMT‐RNR2/PRDX3 interactions in fibroblasts, cells were transfected at approximately 70% confluence with predesigned antisense oligonucleotides (ASOs; Table S8), and RIP assays were performed 24 hours post‐transfection.

To assess endogenous PRDX3 protein turnover, HDFa cells were treated for 2 hours with 0.5 µm MG132 (Sigma–Aldrich, Cat. No. M7449), 5 µg/mL cycloheximide (CHX, Sigma–Aldrich, Cat. No. C4859), 10 µm ubiquitin E1 inhibitor PYR‐41 (Sigma–Aldrich, Cat. No. 662105), 2 µm UbcH13 E2 inhibitor (Sigma–Aldrich, Cat. No. 662107), 10 µm Heclin E3 inhibitor (Sigma–Aldrich, Cat. No. SML1396), and 1 µm Bafilomycin L1 (Sigma–Aldrich, Cat. No. 88899‐55‐2). Following treatment, cells were washed with PBS, and proteins were isolated for Western blot analysis.

### 4.4 Northern Blot

After total RNA purification, RNA was treated with DNase I (ThermoFisher Scientific) as previously described [90], followed by phenol–chloroform extraction. Purified RNA (40 µg) was digested with RNase R (Abcam), according to previously published protocols [91]. Following digestion, RNA samples were purified by phenol–chloroform extraction and ethanol precipitation.

Northern blots were performed according to standard protocols. Briefly, RNA was separated by electrophoresis in a 6% TBE‐Urea gel (ThermoFisher Scientific) in TBE buffer, transferred onto Hybond‐N membranes (Amersham) and UV‐crosslinked [90, 91]. RNA was detected using radiolabelled DNA probe complementary to the target [90]. 30 pmol of DNA probe (circMT‐RNR2_R, Table S8) was end‐labelled using T4 Polynucleotide Kinase (ThermoFisher Scientific) and [γ‐32P]ATP (Revvity), then purified using ProbeQuant G‐50 Micro Columns (Cytiva). After 2‐hour prehybridization of the membrane in hybridization buffer (Church & Gilbert's), the probe was added for an overnight hybridisation at 50°C. Following two washing steps, signals were detected using a Molecular Imager Fx system (Bio‐Rad).

### 4.5 Proliferation and Migration Assays

HDFa cell proliferation and migration were assessed using the IncuCyte live‐cell imaging system (Sartorius, Germany). For the proliferation assay, cells were seeded at low density (typically 20 000 cells/well) in 12‐well plates (Sarstedt, Germany) and allowed to adhere overnight. For the migration assay, ImageLock 96‐well plates (Essen Bioscience, Ann Arbor, MI) were pre‐coated with Collagen I and incubated overnight. HDFa cells were seeded at 15 000 cells per well and allowed to adhere for several hours or overnight. Once a confluent monolayer was formed, a scratch was created using the IncuCyte wound maker (Essen Bioscience). Cells were then imaged every 2 hours, and migration was quantified with the IncuCyte ZOOM software. Proliferation marker MKI67 was measured by qRT‐PCR.

### 4.6 Mitotracker Green and Mitosox Red Staining

After incubation in either high‐ or low‐glucose medium and exposure to hypoxic conditions or following transfection, cells were stained with MitoSOX Red (Thermo Fisher Scientific), a mitochondrial superoxide indicator, at 500 nm for 30 minutes according to the manufacturer's instructions. Subsequently, MitoTracker Green (Thermo Fisher Scientific) was added at 100 nm for 10 minutes. Cells were then washed with HBSS and imaged using a ZEISS confocal microscope at the Biomedicum Imaging Core (BIC), Karolinska Institutet. Fluorescence intensity was quantified using ImageJ.

### 4.7 Mitochondrial Permeability Transition Pore (mPTP) Assay

In an 8‐well chamber slide, 8000 cells were seeded per well. The mitochondial permeability transition pore (mPTP) assay was performed using the Image‐iT LIVE Mitochondrial Transition Pore Assay Kit (I35103, ThermoFisher Scientific), following the manufacturer's instructions. Briefly, stock solutions provided in the kit (1.0 mm Hoechst 33342 and 1.0 m Cobalt (II) chloride hexahydrate) or prepared with DMSO (1.0 mm calcein AM, 200 um MitoTracker Red CMXRos and 500 um ionomycin) were used. To label the cells, a labeling solution was prepared in HBSS containing 1 uL of each of the following stock solutions: 1.0 mm calcein AM stock solution, 200 µm MitoTracker Red CMXRos stock solution, 1.0 mm Hoechst 33342 dye and 1.0 m CoCl2. Cells were covered with the labeling solution and incubated for 15 minutes at 37°C, protected from light. After washing with HBSS, cells were incubated either with 1.0 µm Ionomycin solution in HBSS or with HBSS alone. Images were acquired using a ZEISS confocal microscope at the Biomedicum Imaging Core (BIC), Karolinska Institutet and fluorescence intensity was quantified using ImageJ.

### 4.8 Analysis of Single‐Cell RNA‐Sequencing Data

We analyzed publicly available scRNA‐seq data of diabetic foot ulcers (DFUs) from Theocharidis et al. (GSE165816) [53], including samples from 9 healthy controls and 11 DFU patients. Cells with <500 detected genes, >20% mitochondrial content, or <1,000 total counts were removed. After quality control, we applied our previously described workflow [81], including normalization, identification of highly variable features, batch correction, dimensionality reduction, and unsupervised clustering (resolution 0.8). Cell type annotation was performed based on canonical marker gene expression as previously described [53] and gene expression analyses focused on fibroblast populations.

### 4.9 RNA Pulldown and Mass Spectrometry

The circMT‐RNR2 pulldown assay was conducted using biotin‐labeled probes and streptavidin‐coated magnetic beads. A probe specific to circMT‐RNR2 was designed, with a control probe included to assess specificity. Pulldown assay was carried out with the Pierce Co‐Immunoprecipitation (Co‐IP) Kit (Thermo Fisher Scientific) according to the manufacturer's instructions. The captured RNA‐protein complexes were eluted and bound proteins were analyzed by mass spectrometry at the Proteomics Biomedicum Core Facility (Karolinska Institutet). The mass spectrometry proteomics data have been deposited to the ProteomeXchange Consortium via the PRIDE [92] partner repository with the dataset identifier PXD067605 (Token: htLz3AQaMKxK). The associated RNAs were extracted using TRIzol and analyzed by qRT‐PCR to confirm enrichment of circMT‐RNR2.

### 4.10 In Silico Analysis of Interaction Domains in the circMT‐RNR2–PRDX3 RNA–Protein Complex

Chai‐1, an AlphaFold3‐based structure prediction framework, was used to predict the 3D structure of the circMT‐RNR2–PRDX3 RNA–protein complex [65]. The amino acid sequence of PRDX3 was retrieved from the UniProt database, and the nucleotide sequence of circMT‐RNR2 was obtained based on its validated back‐splice junction. Both sequences were submitted to Chai‐1 for RNA–protein complex structure prediction.

The predicted complex structures were subsequently visualized and analyzed using PyMOL [66]. To identify atomic‐level contacts and putative binding regions within circMT‐RNR2, PyMOL contact analysis tools, including the “find polar contacts” and “find any atoms” functions, were applied to detect interactions between PRDX3 residues and circMT‐RNR2 nucleotides.

### 4.11 Combination of Fluorescent In Situ Hybridization (FISH) and Immunofluorescence Analysis

Fluorescent in situ hybridization (FISH) was performed as previously described [93]. Briefly, a digoxigenin‐labeled single‐stranded DNA oligonucleotide probe targeting the back‐splice junction of circMT‐RNR2 (Table S8) was designed and synthesized by Integrated DNA Technologies. Cells were fixed with 4% paraformaldehyde, permeabilized with 0.3% Triton X‐100 in PBS, and blocked in PBS containing 0.1% Tween‐20 and 10% BSA. Hybridization was performed overnight. Following hybridization, samples were incubated with an anti‐digoxigenin–HRP antibody, and signal amplification was achieved using Alexa Fluor 647 tyramide reagent (Life Technologies). Immunofluorescence staining was subsequently carried out to assess the subcellular localization of PRDX3 using an anti‐PRDX3 antibody (Table S8). Finally, samples were mounted with SlowFade Diamond Antifade Mountant containing DAPI (Life Technologies).

### 4.12 RNA Immunoprecipitation

RNA immunoprecipitation (RIP) was performed using the Magna RIP RNA‐Binding Protein Immunoprecipitation Kit (Millipore, Burlington, MA). HDFa cells were lysed in RIP lysis buffer, and 100 µL of the whole‐cell extract was incubated with an anti‐human PRDX3 antibody (ab222807, Abcam) conjugated to Protein A + G magnetic beads (Millipore) in RIP buffer. Normal rabbit IgG (Millipore) was used as a negative control. After incubation, proteinase K treatment was applied to digest proteins, and the immunoprecipitated RNA was isolated. The levels of circMT‐RNR2 were then quantified by qRT‐PCR.

### 4.13 Mouse Skin Specimens and In Vivo Wound Model

Mouse skin samples were collected from Eleven‐week‐old male C57BL/6J (wild‐type) mice or diabetic db/db [BKS(D)‐Lepr^db^/JOrlRj] mice on the C57BL/6J background (Janvier Labs, Le Genest‐Saint‐Isle, France) as we previously reported [94]. Briefly, each mouse received 4‐mm excisional wounds, and the excised skin served as an intact skin control. Wound‐edge tissues were collected using a 6‐mm punch at 3, 7, and 10 days post‐wounding (DPW). The samples were then used for RNA extraction and qRT‐PCR analysis. For dermis and epidermis separation, samples were washed twice with PBS to remove debris and incubated overnight at 4°C with Dispase II (5 U/mL; Roche). The whole skin as well as the sperated dermis and epidermis were used for RNA extraction and qRT‐PCR analysis. For cell isolation, the dermal tissue was minced into small fragments before enzymatic digestion at 37°C for 3–4 hours using the Whole Skin Dissociation Kit (Miltenyi Biotec, 130‐101‐540). The resulting suspension was filtered through a 70‐µm mesh and centrifuged to obtain single cells. Fibroblasts were purified using the PDGFRα MicroBead Kit (Miltenyi Biotec, 130‐101‐502) following the manufacturer's protocol, and the isolated cells were used for RNA extraction and qRT‐PCR analysis.

An in vivo wound healing model was established using 8–9 weeks old male mice with C57BL/6 J background over a seven‐day period. The dorsal area of each mouse was shaved and treated with depilatory cream to remove hair. Two 4 mm full‐thickness wounds were created on the dorsal surface of each mouse. A total of 5 µL of treatment, containing either 0.1 nmol si‐CircMT‐RNR2 (n = 5) or si‐Control (n = 5), along with in vivo‐jetPEI (Cat. 201–10 G, Polyplus‐transfection, France), were applied topically at two time points: Day 0 and Day 2. The wounds were covered with Tegaderm film dressings. Pain relief was provided via intramuscular administration of Temgesic (0.003 mg/mL) at a dose of 100 µL per 10 g of body weight on Day 0 and Day 1. Wound images were captured on Days 0, 2, 4, and 6 to document the healing process. On Day 6, mice were euthanized in a CO_2_ chamber, and wound tissues were harvested using a 6 mm biopsy punch. The collected biopsies were used for RNA extraction and qRT‐PCR analysis to assess gene expression, as well as for histological examination via H&E staining. All animal procedures were approved by the Swedish Animal Ethics Review Board (Dnr. 11854–2020) and conducted in accordance with relevant guidelines and regulations.

### 4.14 Human Ex Vivo Wound Model

Skin from discarded surgical procedures was cleaned with 70% ethanol, and partial‐thickness wounds (restricted to the dermis) were created using a 2 mm biopsy punch. These wounds were then excised with a 6 mm punch and placed in 8‐well chamber plates for in vitro culture. Samples were maintained in DMEM supplemented with 10% FBS, 1% penicillin–streptomycin, and 1% antifungal agent (Thermo Fisher Scientific). 80 µL of medium was added to each biopsy, ensuring the epidermis remained exposed to air to preserve a liquid–air interface. Cultures were incubated at 37°C in a humidified 5% CO_2_ atmosphere.

For treatments, si‐CircMT‐RNR2 or si‐Ctrl (n = 3 per condition) was applied topically at 1.5 µg/µL, mixed with 10% glucose, PBS, and JetPei transfection reagent (Polyplus‐transfection). CircMT‐RNR2 overexpression (OE) and control plasmids were applied at 3 µg/µL. A total of 10 µL treatment was administered on Days 0 and 2. Wound samples were collected six days post‐injury for RNA extraction and qRT‐PCR to evaluate transfection efficiency and gene expression. Wound closure was assessed using CellTracker Green CMFDA Dye (Invitrogen). Briefly, 4 µL dye (50 µm) was added to each tissue and incubated at 37°C with 5% CO_2_ for 30 minutes. After PBS washing, wounds were imaged with a Nikon Eclipse Ni‐E fluorescence microscope, and wound areas were quantified using ImageJ.

### 4.15 Statistics

The number of biological replicates for each experiment is specified in the respective methods sections and figure legends. Comparisons between two groups were made using either a paired or unpaired Student's t‐test, while comparisons involving multiple groups were analyzed using one way or two‐way analysis of variance (ANOVA).Comparisons between single cell samples were achieved using Mann–Whitney U test. Statistical significance was determined at a threshold of p < 0.05. Statistical analyses were conducted using GraphPad Prism software version 9 and R software version 4.3.3.

## Author Contributions

NXL, GN, JG, AW, PS, DL, EL and XZ conceived the study and designed the experiments. GN, JG, YX, LP, and MG collected clinical samples and performed the experiments. GN and JG analyzed the data and prepared the figures. YC, ZL, LL conducted bioinformatic analysis. NXL, GN and JG collaboratively prepared the original manuscript draft with the help from all authors.

## Conflicts of Interest

The authors declare no conflicts of interest.

## Supporting information




**Supporting File 1**: advs74271‐sup‐0001‐SuppMat.docx.

**Supporting File 2**: advs74271‐sup‐0002‐Table S1 Human sample information.xlsx.

**Supporting File 3**: advs74271‐sup‐0003‐Table S2 DEG in HDFa with circMT‐RNR2 knockdown.xlsx.

**Supporting File 4**: advs74271‐sup‐0004‐Table S3. KEGG pathway analysis of DEG in HDFa with circMT‐RNR2 knockdown.xlsx.

**Supporting File 5**: advs74271‐sup‐0005‐Table S4. Transcription factor enrichment analysis of downregulated genes in HDFa with circM 5T‐RNR2 knockdown.xlsx.

**Supporting File 6**: advs74271‐sup‐0006‐Table S5 FOXM1 regulated genes in in HDFa with circMT‐RNR2 knockdown.xlsx.

**Supporting File 7**: advs74271‐sup‐0007‐Table S6 Metabolic pathway analysis of DEG in HDFa with circMT‐RNR2 knockdown.xlsx.

**Supporting File 8**: advs74271‐sup‐0008‐Table S7. Mass spectrometry analysis of protein interactome of circMT‐RNR2.xlsx.

**Supporting File 9**: advs74271‐sup‐0009‐Table S8. List of reagents used in this study_clean.xlsx.

## Data Availability

The data that support the findings of this study are available from the corresponding author upon reasonable request.
